# Functional/dissociative seizures: Progress not perfection

**DOI:** 10.1111/epi.70033

**Published:** 2025-11-26

**Authors:** Kette Valente, Colin Reilly

**Affiliations:** ^1^ University of São Paulo Medical School São Paulo Brazil; ^2^ Institute of Neuroscience and Physiology, Sahlgrenska Academy University of Gothenburg Gothenburg Sweden

To the Editors

We commend Hingray and colleagues for their timely and thoughtful proposal to adopt the label functional/dissociative seizures (FDS) as a replacement for psychogenic nonepileptic seizures.^1^ This work represents an advance in addressing the limitations and stigma of previous terminology. The authors should be congratulated for providing a comprehensive, inclusive, and patient‐centered framework.

## STRENGTHS OF THE CURRENT PROPOSAL

The primary strength of FDS is its alignment with both Diagnostic and Statistical Manual of Mental Disorders, 5th Edition (functional neurological symptom disorder) and International Classification of Diseases, 11th Revision (ICD‐11; dissociative neurological symptom disorder).^2^, ^3^ It will facilitate international coding, billing, and research comparability and collaboration. The emphasis on stigma reduction, supported by consultation with advocacy organizations, is particularly welcome. Importantly, the move away from “psychogenic” and “nonepileptic” avoids longstanding pejorative and negative connotations that have hindered patient acceptance.

The integrative definition reflects the biopsychosocial nature of these seizures, recognizing paroxysmal semiology, impaired consciousness, and autonomic activation. The approach helps distinguish FDS from panic attacks, malingering, or epileptic seizures, while preserving the patient‐reported reality of seizurelike experiences.

The decision to retain “seizure” respects survey data showing that patients find this term more validating than “attack” or “event.”^4^ It also communicates clinical seriousness to health professionals and fosters acceptance of treatment plans if used carefully.

## REMAINING CHALLENGES

Using a slash—“functional/dissociative”—offers flexibility but also risks reinforcing ambiguity. For instance, neurologists may prefer functional, whereas psychiatrists may favor dissociative. Therefore, clinicians may privilege one term over the other, potentially deepening regional and disciplinary divides rather than fostering unified practice.^4^, ^5^, ^6^

Each term has its own limitations. Functional conveys reversibility and consistency with other medical terminology but remains nonspecific and sometimes confusing to patients. Dissociative aligns with ICD nosology and psychotherapy but may not resonate with those lacking dissociative features or trauma histories. Both terms, therefore, risk achieving only partial acceptability. The use of the word seizure may remain problematic in certain cultures and languages.

## PEDIATRIC CONSIDERATIONS

In the earlier International League Against Epilepsy (ILAE) consensus‐based recommendations developed by the Task Force on Pediatric Psychiatric Issues, experts representing five ILAE regions did not endorse the use of the term dissociative in children.^7^

Nevertheless, it is important to recognize that dissociative terminology does appear in pediatric psychiatry—particularly in the context of anxiety and somatoform presentations—and may hold descriptive value when differentiating FDS from other nonepileptic phenomena.^7^, ^8^ The pediatric context, however, remains distinct. Children and adolescents often have different predisposing factors, semiological features, and neurodevelopmental vulnerabilities that may not map neatly onto adult frameworks.^7^

Accordingly, the dichotomy between functional and dissociative may require nuanced application across developmental stages rather than a categorical distinction. Terminology should be developmentally sensitive, family‐oriented, and accompanied by age‐appropriate communication strategies. Further research and direct engagement with young people and families are needed to ensure that the FDS framework achieves broad acceptance in pediatric settings.

## GLOBAL APPLICABILITY

The Task Force on Pediatric Psychiatric Issues rightly notes the difficulty of achieving universal global consensus. Although strongly recommended by the ILAE, representation from low‐ and middle‐income countries in the process was limited, even though these regions carry a substantial seizure burden. Cultural interpretations of seizures and linguistic challenges in translating functional or dissociative may limit the term's resonance in some contexts. Ongoing cross‐cultural engagement will be crucial to ensure international acceptability.

## BROADER PERSPECTIVE

The question remains: Do we have a single term that can fully encompass the heterogeneity of this condition across ages, cultures, and clinical presentations? Probably not.

Over the years, we have observed the ILAE's painful struggle with the words awareness and consciousness.^9^, ^10^ The use of specific nomenclature that is not broadly accepted can compromise scientific precision, clinical usability, and patient acceptability. Consensus on terminology is therefore greatly needed.

The value of the FDS proposal lies not in perfection but in progress. By providing a pragmatic and less‐stigmatizing foundation, the FDS framework advances communication, service development, and research while opening the door to further refinement.

In conclusion, the Task Force on Pediatric Psychiatric Issues delivered a critical step forward by replacing outdated terminology with a patient‐centered, biopsychosocial, and internationally anchored framework. Limitations remain—notably the dual label, risks of semantic ambiguity, pediatric applicability, and cross‐cultural resonance—but these challenges reflect the condition's inherent complexity rather than flaws in the process. We applaud the authors' leadership in unifying a fragmented field and look forward to further debate, empirical validation, and adaptation across developmental and cultural contexts.

## CONFLICT OF INTEREST STATEMENT

Neither of the authors has any conflict of interest to disclose. We confirm that we have read the *Epilepsia* and International Committee of Medical Journal Editors statements on ethical publication and affirm that this report is consistent with those guidelines.

## Data Availability

Data sharing not applicable to this article as no datasets were generated or analysed during the current study.
